# Tracing amino acid exchange during host-pathogen interaction by combined stable-isotope time-resolved Raman spectral imaging

**DOI:** 10.1038/srep20811

**Published:** 2016-02-09

**Authors:** Abida Naemat, Hany M. Elsheikha, Radu A. Boitor, Ioan Notingher

**Affiliations:** 1 School of Physics and Astronomy, University of Nottingham, Nottingham, NG7 2RD, UK; 2 Faculty of Medicine and Health Sciences, School of Veterinary Medicine and Science, University of Nottingham, Sutton Bonington Campus, Leicestershire, LE12 5RD, UK

## Abstract

This study investigates the temporal and spatial interchange of the aromatic amino acid phenylalanine (Phe) between human retinal pigment epithelial cell line (ARPE-19) and tachyzoites of the apicomplexan protozoan parasite *Toxoplasma gondii* (*T. gondii*). Stable isotope labelling by amino acids in cell culture (SILAC) is combined with Raman micro-spectroscopy to selectively monitor the incorporation of deuterium-labelled Phe into proteins in individual live tachyzoites. Our results show a very rapid uptake of l-Phe(D8) by the intracellular growing parasite. *T. gondii* tachyzoites are capable of extracting l-Phe(D8) from host cells as soon as it invades the cell. l-Phe(D8) from the host cell completely replaces the l-Phe within *T. gondii* tachyzoites 7–9 hours after infection. A quantitative model based on Raman spectra allowed an estimation of the exchange rate of Phe as 0.5–1.6 × 10^4^ molecules/s. On the other hand, extracellular tachyzoites were not able to consume l-Phe(D8) after 24 hours of infection. These findings further our understanding of the amino acid trafficking between host cells and this strictly intracellular parasite. In particular, this study highlights new aspects of the metabolism of amino acid Phe operative during the interaction between *T. gondii* and its host cell.

*Toxoplasma gondii* is the most prevalent protozoal infection of the Central Nervous System. It causes a spectrum of human and animal diseases ranging from abortion to neurological disorders^1^. This intracellular apicomplexan protozoan parasite has the capacity to infect all vertebrate species within virtually any phagocytic and nonphagocytic cell type. It survives within its own membrane-bound compartment referred to as the parasitophorous vacuole (PV)^2^ The broad range of cells amenable to infection by *T. gondii* reflects the plasticity of the parasite’s metabolism and versatility in scavenging nutrients to support its intracellular growth^3^,^4^. Nutrient acquisition from the host is a strategic utilization of essential nutrients, such as amino acids (AA), lipids, and glucose to support the parasite’s metabolic requirements. *T. gondii* and other apicomplexans are fast-replicating organisms and thus are critically dependent on host-cell nutrients as carbon and energy sources for the extensive membrane biogenesis required during their replication^5^.

Even though *T. gondii* can synthesise some AA *de novo*, this parasite lacks synthetic pathways for some AA, which must be retrieved from the host cells^6^,^7^,^8^,^9^. Therefore, the pools of amino acids in the host’s cytosol constitute an important reservoir to complement the endogenous production of AAs in the parasite. For example, the replication rate of *T. gondii* is proportional to the concentration of tryptophan in the host cell^10^,^11^.

Phenylalanine (Phe) is one of the most vital AA needed by both mammalian cells and *T. gondii*. The *T. gondii* genome encodes hydroxylase enzymes, which can metabolize both Phe and Tyrosine (Tyr) to l-DOPA, with a substrate preference for Tyr^12^. However, little is known regarding the transport of Phe from mammalian cell hosts to the parasite, the formation of precursor pool, protein synthesis, or protein turnover within the parasite. Unravelling these complex processes requires novel approaches that are capable of monitoring the shuttling of AA from or toward the host during *T. gondii* infection.

Isotopically labelled AA tracer is frequently used to trace the kinetics of incorporation of a labelled AA into newly synthesized proteins. It can also be used to characterize the acquisition of AA by the growing parasite. Various isotope labelling approaches exist for studying protein dynamics and turnover. These methods include stable isotope labelling by amino acids in cell culture (SILAC), chemical derivatization (GC-MS, ICAT, iTRAQ, TMT), and enzymatically catalyzed incorporation (^18^O labelling). However, these approaches are invasive, cost- and/or time-prohibitive, and might not be amenable to the biological system of interest. Also, these methods cannot elucidate the kinetic events that occur rapidly and change from cell to cell. Thus, these techniques alone cannot quantitatively describe the complex dynamic interactions between engaging host and parasite systems.

Raman micro-spectroscopy (RMS) is a non-invasive, label-free imaging technique that can enable non-invasive molecular analysis of dynamic events in live cells *in-vitro*^13^. Its utility has been confirmed in studies covering a wide spectrum of applications, including cellular differentiation^14^,^15^,^16^, apoptosis^17^,^18^,^19^, drug-cell interactions^20^. Also, previous studies have demonstrated the ability of RMS to detect molecular changes during parasite development within host cells^21^. However, this approach could not provide enough specificity to reveal which molecules (e.g., nucleotides, glucose, lipids or amino acids) are altered during host-*T. gondii* interaction.

Combining stable-isotope labelling with RMS can increase the chemical specificity of Raman bands, and identify the origin of the molecular species and monitor its metabolism^22^,^23^,^24^,^25^,^26^,^27^. In this study, we show for the first time that it is possible to combine time-resolved RMS and stable-isotope labelling approach to monitor the exchange of molecules between individual cells in real-time. We monitor the kinetic of labelled Phe acquisition by *T. gondii* from the host cell during the first 48 hours of the parasite’s intracellular development. Identification of *T. gondii* metabolic requirements during infection provides insight into the nutritional cues being sensed by the parasite and could reveal unique targets for novel therapeutics.

## Results

### Host cell uptake of l-Phe(D8)

First, we investigated the rate at which l-Phe(D8) tracer replaces l-Phe within the host cells. ARPE-19 cells were metabolically labelled by adding l-Phe(D8) to customised Phe-free DMEM medium. The only source of Phe for the cells is the customised culture medium. The uptake of l-Phe(D8) is expected to be driven mostly by new protein biosynthesis during cell division and protein turnover in non-dividing cells. The doubling time of ARPE-19 cells grown *in vitro* was determined using cell counting at a series of 7 time points collected over a period of 7 days, revealing a doubling time of 24 hours. To achieve complete substitution of l-Phe(D8), ARPE-19 cells were maintained in serum-free DMEM overnight. Then, cells were maintained in complete DMEM with serum, but free of any Phe for 24 hours. The culture medium was then replaced by l-Phe(D8)-supplemented medium.

**Figure 1:**
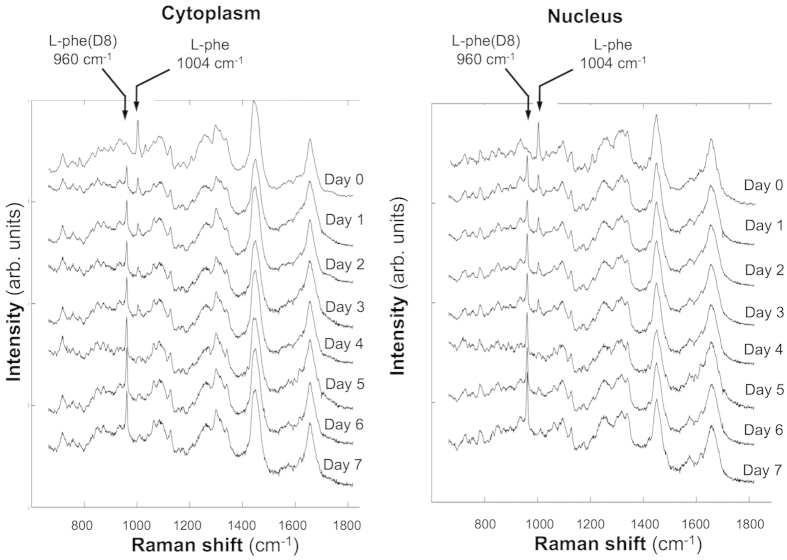
Time-course Raman spectra showing l-Phe(D8) uptake by growing human retinal cells (ARPE-19 cells). Raman spectra were measured in the cytoplasm and nucleus of ARPE-19 cells cultured in **l-**Phe(D8)-containing medium, after fixation in 4% paraformaldehyde and washing in PBS, at the indicated time points. Spectra are shifted vertically for clarity.

Figure 1 shows the average Raman spectra measured from the cytoplasm and nucleus of ARPE-19 cells growing in l-Phe(D8)-containing medium. The spectra in Fig. 1 were obtained from cells fixed with 4% paraformaldehyde and washed with PBS. This treatment maintained the morphology of the cells and ensured that the Raman spectra were not contaminated by signals from the culture medium,thus allowing discrimination between spectra of cytoplasm and nucleus. Prior to supplementing the culture medium with l-Phe(D8), the sharp Raman band corresponding to the ring-breathing mode of l-Phe could be identified at 1004 cm^−1^. After using culture medium containing l-Phe(D8) the frequency of this vibrational mode shifted to 960 cm^−1^. Figure 1 shows the time-dependent increase in the intensity of the 960 cm^−1^ Raman band corresponding to l-Phe(D8). This coincides with a decrease in the intensity of the 1004 cm^−1^ band, which corresponds to l-Phe. After one doubling time (1 day), the ratio **I**_960_/(**I**_960_ + **I**_1004_) is ~0.53 for the spectra measured in the cytoplasm and ~0.60 in the nucleus, indicating a similar distribution of newly synthesized proteins in the host cells. The substitution increases with time, as the ratio **I**_960_/(**I**_960_ + **I**_1004_) in both the cytoplasm and nucleus becomes ~0.72 at 4 days, and reaches 0.9–1.0 at days 5. At this point, the Raman band at 1004 cm^−1^ cannot be detected.

**Figure 2:**
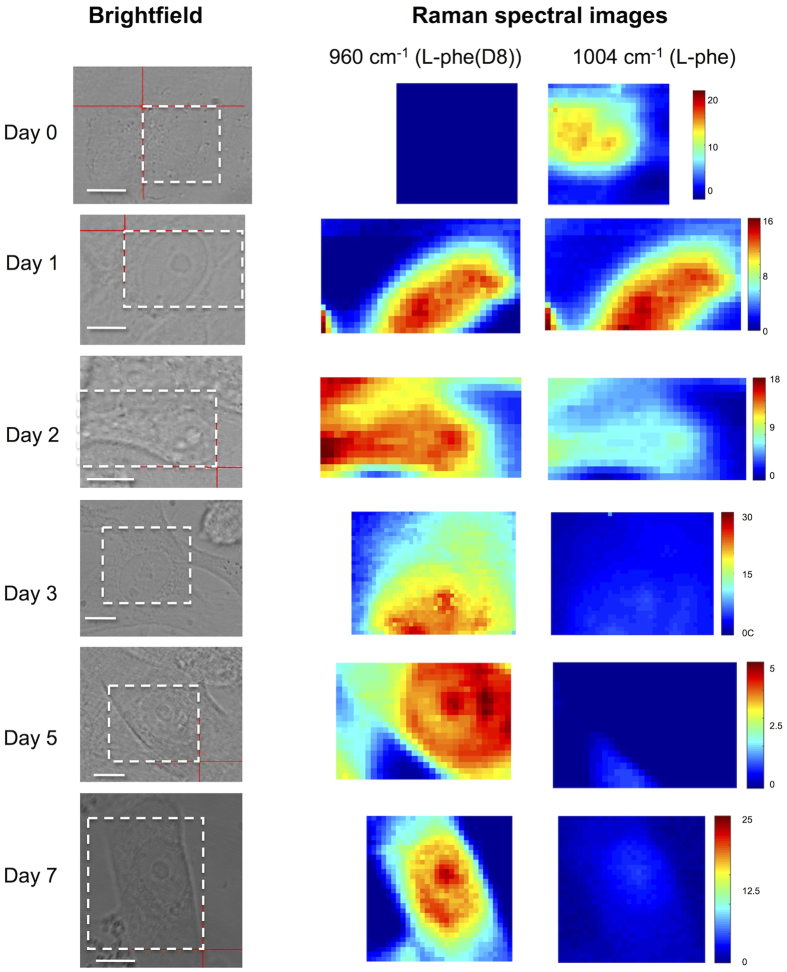
Time-course Raman spectral maps of fixed human retinal cells showing cellular incorporation l-Phe(D8). Raman spectral images were obtained by plotting the height of Raman bands assigned to the ring-breathing mode vibrations of **l-**Phe (1004 cm^−1^) and **l-**Phe(D8) (960 cm^−1^). Cells were grown in **l-**Phe(D8)-supplemented medium, fixed in 4% paraformaldehyde and washed in PBS prior to Raman measurements. The areas scanned by Raman spectroscopy are highlighted by white dashed-line rectangles (the red crosses should be ignored). *Scale bars*: 10 μm.

Raman micro-spectroscopy (RMS) was also used to evaluate the intracellular distribution and accumulation of l-Phe(D8) over time (Fig. 2). After short incubation times in l-Phe(D8) medium, the Raman spectral maps corresponding to the 1004 cm^−1^ and 960 cm^−1^ bands indicate similar patterns for the distribution of l-Phe and l-Phe(D8), supporting their biological equivalence. Comparison between the Raman spectra recorded from locations in the cell cytoplasm and nucleus indicates that the incorporation of l-Phe(D8) occurs at the similar rates.

**Figure 3:**
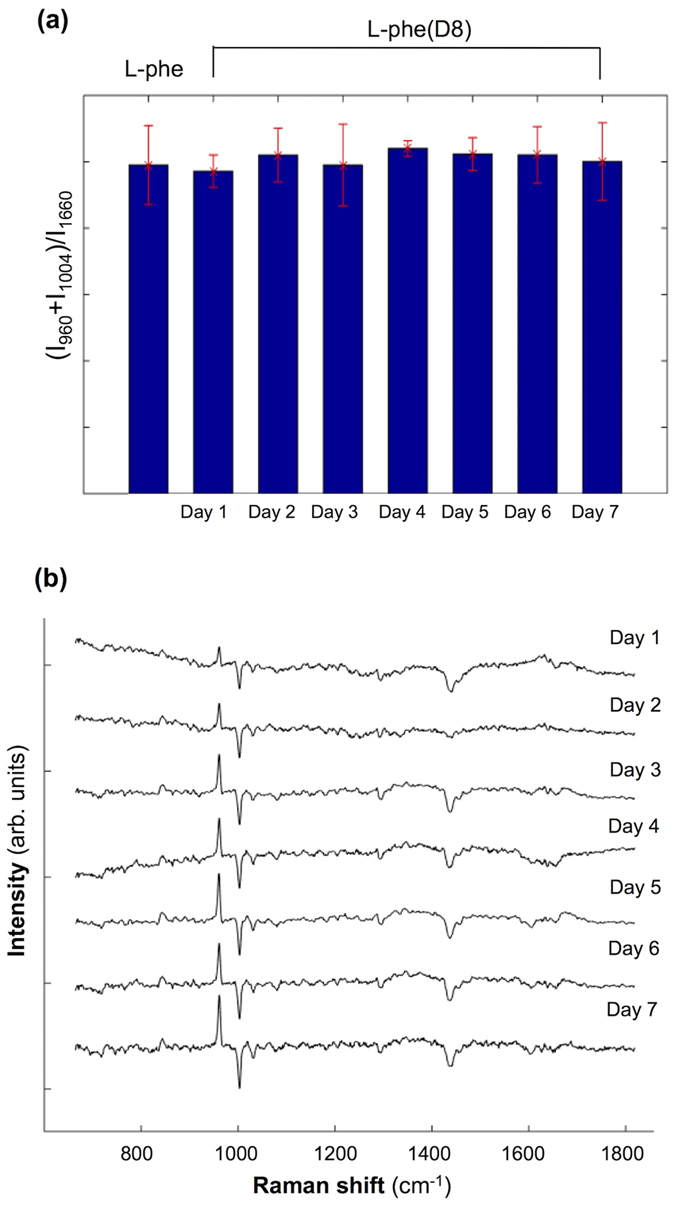
Potential detection of metabolic products in cells following cellular uptake of l-Phe(D8). (**a**) Calculated ratio (**I**_1004_ + **I**_960_)/**I**_1660_ for ARPE-19 cells grown in **l-**Phe and **l-**Phe(D8) culture media. (**b**) Computed difference Raman spectra acquired from cells cultured in **l-**Phe and **l-**Phe(D8) daily for 7 days. Spectra are shifted vertically for clarity.

While the 1004 cm^−1^ and 960 cm^−1^ Raman bands are assigned to ring-breathing vibrations of l-Phe and l-Phe(D8), respectively, the intensity of the 1660 cm^−1^ Raman band corresponds mainly to the Amide I vibrational modes, with some contributions from C = C vibrations in unsaturated lipids. Thus, the intensity of this band can be related to the overall level of protein synthesis. Thus, the ratio between the band intensities of the ring-breathing modes of Phe (**I**_960_ + **I**_1004_) and the Amide I (**I**_1660_) can be linked to the level of incorporation of Phe into proteins. Figure 3a shows the calculated values of the ratio (**I**_960_ + **I**_1004_)/**I**_1660_ for cells grown *in-vitro* in culture media containing l-Phe and l-Phe(D8) at different time points. Regardless of the incubation time, the uptaken l-Phe-(D8) is incorporated into proteins at similar levels as l-Phe under normal culture conditions.

To determine whether the uptaken l-Phe(D8) is also metabolized by the ARPE-19 cells, the difference between the average Raman spectra for cells cultured in medium containing l-Phe and l-Phe(D8) were calculated (see Fig. 3a). The results indicate that apart from the downshift of the ring-breathing mode of Phe from 1004 cm^−1^ to 960 cm^−1^, no other major changes can be observed that could be related to the isotope substitution.

Of particular interest is the absence of changes associated with tyrosine (Tyr), a metabolic product of Phe. Typically, in the Raman spectra of cells, Tyr can be identified as a spectral doublet at 828 cm^−1^ and 854 cm^−1^, which corresponds to a Fermi resonance between the ring-breathing mode and the overtone of the out-of-plane ring-bending mode^28^. Upon substitution of H with D in Tyr, the frequency of the out-of-plane ring-bending mode was found to drop to 362 cm^−1^ (with an overtone at 724 cm^−1^) while the ring-breathing mode occurs at ~791 cm^−1 ^28. Because of the difference in the frequencies for these two vibrational modes, the Fermi resonance is removed and only a single peak at ~791 cm^−1^ can be observed in the Raman spectrum of Tyr(D4). The presence of this peak in Raman spectra from cells incubated in l-Phe(D8) medium would indicate conversion of l-Phe(D8) into Tyr(D7). The calculated difference spectra in Fig. 3a show no changes in the Raman bands in the 770–790 cm^−1^, but rather an increased peak at ~840 cm^−1^. It is also important to mention that the detection of the 791 cm^−1^ band of Tyr(D7) may be hindered by the overlap with the Raman bands corresponding to nucleic acids (788 cm^−1^ O-P-O phosphodiester stretching vibrations). Therefore, no conversion of l-Phe(D8) into Tyr(D7) can be detected. These results indicate that the l-Phe(D8) is directly incorporated into newly synthesized proteins.

### Differential uptake of l-Phe(D8) by extracellular versus intracellular *T. gondii*

**Figure 4:**
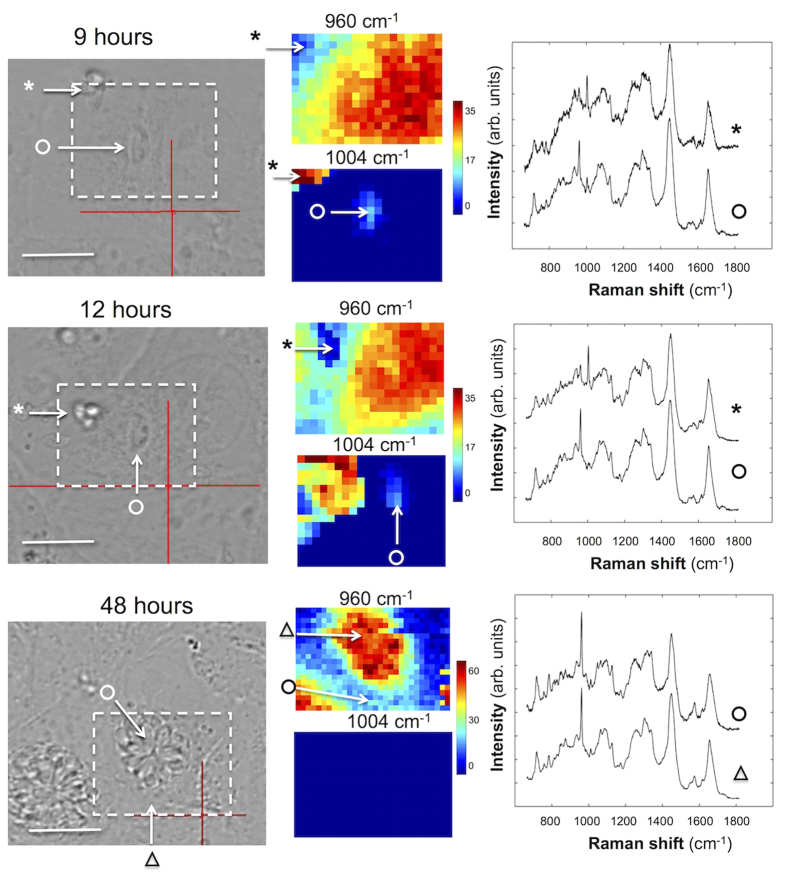
Comparison between Raman spectra of live T. gondii tachyzoites within host ARPE-19 cells (indicated by symbol “o”) and live T. gondii that did not enter host cells and remained extracellular (indicated by “*”). Here the cells were in culture medium containing **l-**Phe(D8). The Raman spectra show rapid uptake of **l-**Phe(D8) by the intracellular *T. gondii*, while no uptake of **l-**Phe(D8) is observed for the extracellular *T. gondii*. At 12 h parasites start to divide (middle) and at 48 h, a large parasitophorous vacuole full of parasites can be observed (bottom) (indicated by symbol “o”). All spectra are shifted vertically for clarity. *Scale bars*: 10 μm.

We know that *T. gondii* tachyzoites are incapable of replication while outside mammalian cells. But, we do not know whether extracellular *T gondii* tachyzoites are able to acquire Phe directly from the culture media. Hence, it is important to confirm that the information provided by the Raman spectra reflects solely molecular exchange between *T. gondii* and host cells and that there is no contribution from the culture medium. In these experiments, the infection was carried out while maintaining the cells in culture medium containing l-Phe(D8). Figure 4 compares the Raman spectra for *T. gondii* infecting ARPE-19 cells and extracellular *T. gondii* tachyzoites that have not yet invaded the host ARPE-19 cells. At 9 hours post infection (PI), individual *T. gondii* tachyzoites were observed inside ARPE-19 cells. Raman spectra indicated a high intensity band at 960 cm^−1^ assigned to l-Phe(D8) while the corresponding band at 1004 cm^−1^ corresponding to l-Phe had decreased by about 4-fold. At 12 hours the 1004 cm^−1^ band could be detected in the spectra of *T. gondii*. These results indicate a very rapid uptake of l-Phe(D8) by *T. gondii* even before the start of the parasite division (occurs at approximately12 hours PI).

In contrast, the Raman spectra of *T .gondii* that did not enter the ARPE-19 cells (marked by * in Fig. 4), contain intense Raman bands at 1004 cm^−1^ corresponding to l-Phe and only small peaks (~5-fold lower) at 960 cm^−1^, both at 9 hours and 12 hours. The lack of change in either the 1004 cm^−1^ or 960 cm^−1^ bands from 9 hours PI to 12 hours PI indicates that this peak is likely the contribution of l-Phe(D8) molecules in the underlying ARPE-19 cell. These results indicate that *T. gondii* cannot uptake Phe directly from the culture medium and that the Raman bands at 960 cm^−1^ identified in the intracellular *T. gondii* (marked by o in Fig. 4) originate from l-Phe(D8) acquired only from the host ARPE-19 cells after infection.

The images recorded at 48 hours PI also show that *T. gondii* developed and replicated successfully as the parasitophorous vacuole (PV) contained many tachyzoites. No differences were observed in the growth of *T. gondii* infecting ARPE-19 cells cultured in l-Phe or l-Phe(D8). These results show that the substitution of l-Phe with l-Phe(D8) and the exposure to 785 nm laser light during Raman spectral measurements did not adversely affect the viability of the cells.

### Time- and spatially-resolved exchange of proteins between host and pathogen

To obtain detailed information regarding metabolism and exchange of Phe between the host ARPE-19 cells and *T. gondii*, time-resolved Raman spectral mapping of *T. gondii* was performed from 1 hour to 48 hours PI. For these experiments, the culture medium was replaced with l-Phe(D8)-free media to avoid any false labelling of the parasite while in the extracellular medium before cell invasion.

**Figure 5:**
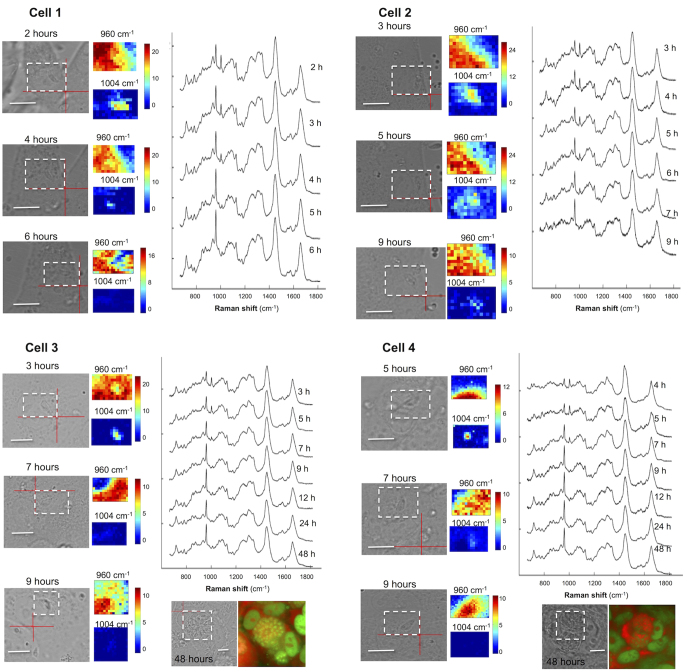
Typical time-course Raman spectra and maps corresponding to the 1004 cm−1 (l-Phe) and 960 cm−1 (l-Phe(D8)) bands of live T. gondii infecting ARPE-1 cells. The measured areas are indicated by the white dashed rectangles. Acridine orange (AO) staining was carried out at 48 h after infection. All spectra are shifted vertically for clarity. *Scale bars*: 10 μm.

Figure 5 presents the time resolved Raman spectra and spectral maps corresponding to the 960 cm^−1^ and 1004 cm^−1^ of four live *T. gondii* tachyzoites during the invasion of ARPE-19 cells. Because the intensities of Raman bands are affected by both molecular and morphological changes of the cells, the spectral maps provide only qualitative information. Nevertheless, at positions corresponding to *T. gondii*, an intense Raman band assigned to l-Phe(D8) at 960 cm^−1^ can be observed at as early as 2 hours PI. A corresponding decrease in the intensity of the 1004 cm^−1^ band assigned to l-Phe can also be detected. It is also interesting to note that Raman bands at 1004 cm^−1^ can be detected in the cytoplasm of the APRE-19 cells, suggesting release of proteins by *T. gondii* into the host. The intensity of the 960 cm^−1^ band increases with time, indicating a rapid uptake of l-Phe(D8) by *T. gondii*. By 9–12 hours PI, the Raman band at 1004 cm^−1^ is no longer detectable in the Raman spectra of *T. gondii*, suggesting that the initial l-Phe in *T*. *gondii* is completely substituted with l-Phe(D8) acquired from the host cells. Although during this time *T. gondii* grow in size, they divide at ~12 hours PI. By this point, l-Phe has been fully replaced by l-Phe(D8).

### Dynamics of uptake of l-Phe(D8) at single parasite level

**Figure 6:**
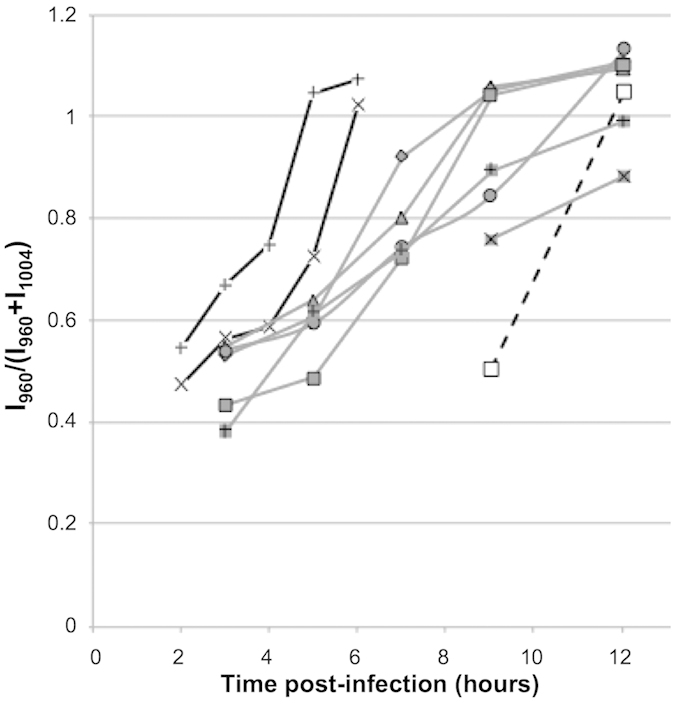
Uptake of l-Phe(D8) by individual T. gondii tachyzoites. Individual tachyzoites were monitored from the moment they invaded the host cells. **l-**Phe(D8) uptake by the tachyzoites was measured by Raman microscopy every hour for 12 hr.

Figure 6 shows the kinetics of Phe exchange between *T. gondii* and the host ARPE-1 cells. The uptake was expressed as the percentage of l-Phe(D8) from the total Phe calculated as the ratio **I**_960_/(**I**_960_ + **I**_1004_). While other techniques require large populations of cells and provide only average results, Raman microscopy can measure the rates of Phe transport for individual live *T. gondii*. Figure 6 shows that more than 90% of *T. gondii* have a rapid uptake of Phe from the host cells, as l-Phe(D8) reached ~50% at 2–3 hours PI. It is interesting to note that after ~3 hours PI, two trends can be observed. For the majority of *T. gondii* the transport rate seems to decrease slightly and the complete substitution of l-Phe with l-Phe(D8) is typically observed at 6–9 hours. For a smaller group of *T. gondii*, the transport rates remain constant and the saturation with l-Phe(D8) is reached as early as 6 hours, which is approximately half the time required for *T. gondii* to divide.

**Figure 7:**
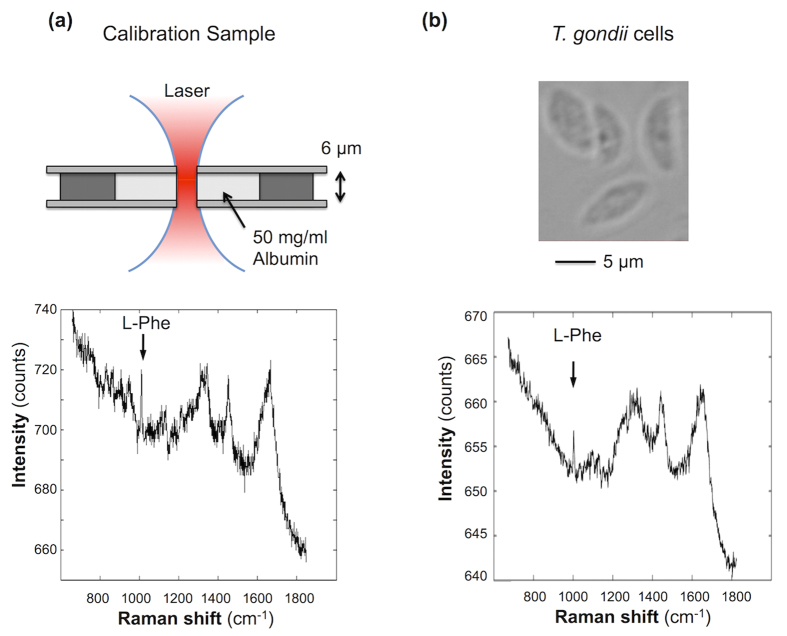
Quantification model for l-Phe(D8) in ARPE-19 cells and T. gondii based on Raman micro-spectroscopy. (**a**) Schematic of the calibration sample of 50 mg/ml human serum albumin in water and the corresponding Raman spectrum. (**b**) Images of isolated *T. gondii* tachyzoites and typical Raman spectrum.

To quantify the transport rates of l-Phe to *T. gondii*, a calibration model was built for the Raman micro-spectrometer based on human serum albumin solution in a sample with known thickness (Fig. 7a). The intensity of the 1004 cm^−1^ Raman band **I**_1004_ assigned to l-Phe is directly proportional to the number of l-Phe molecules **N**_Phe_ irradiated within the sample: **I**_1004_ = ***k*** × **N**_Phe_. For a 50 mg/ml of albumin solution within the cylindrical laser volume (height 6 μm and ~2 μm^2^ area), the number of l-Phe residues is ~1.6 × 10^8^. Based on the intensity of the 1004 cm^−1^ Raman band for the calibration samples (Fig. 7a), the calibration constant was calculated to be ***k*** = 1.2 ± 0.1 × 10^−7^ counts/molec.

Although it was not possible to measure the exact height of the cells used in the time-course experiment described in Figs 5 and 6, the size of *T. gondii* was estimated to be ~2 μm thickness and 8 μm length based on the bright field microscopy images. Using the height of the 1004 cm^−1^ band in the measured Raman spectra for isolated *T. gondii* (Fig. 7b) and an estimated height of ~2 μm, the concentration of l-Phe molecules in *T. gondii* was estimated to be 10 ± 4 × 10^6^ molecules/μm^3^. For a typical value of ~8 μm for the length of *T. gondii*, the total number of Phe molecules in a *T. gondii* was estimated to be 2.6 ± 1 × 10^8^. Based on the results in Fig. 6 indicating that all l-Phe are replaced by l-Phe(D) within 7–9 hours PI, the transport rate of l-Phe(D8) from the host cells to the *T. gondii* is 0.5–1.6 × 10^4^ molecules/s.

## Discussion

Even though phenylalanine (Phe) is a vital component of proteins in all living organisms, the sources of this aromatic amino acid (AA) are organism-dependent. *T. gondii* can synthesize Phe through the shikimate pathway given an adequate supply of a specific precursor, phenylpyruvate, from infected host cell^6^. Despite the capacity for *de novo* synthesis, *T. gondii* still scavenges Phe and other amino acids, which are essential for the host^9^.

In this study, we leveraged the capabilities of a combined stable-isotope labelled Phe and Raman micro-spectroscopy (RMS) approach to investigate the transport of Phe from host cell to live *T. gondii*, both in time- and spatial-domains. Specifically, we examined the relationship among the processes of Phe transport, possible pool build up, and the incorporation of Phe into newly synthesized protein. Our findings revealed that the time taken for the transport of l-Phe(D8) to *T. gondii* was remarkably quick. *T. gondii* tachyzoites acquired l-Phe(D8) from host cells at a very rapid rate, ~50% at 2–3 hours post infection (PI), and l-Phe became completely substituted with l-Phe(D8) at ~7–9 hours PI, which even precedes parasite division.

Uptake of l-Phe(D8) might consist of active transport through transporters in the parasite membrane into a free AA internal pool. The Raman spectral results indicated that the uptaken l-Phe(D8) was not metabolized/cleaved into tyrosine (Tyr) by the parasite. These results suggest that l-Phe(D8) uptaken by *T. gondii* might be incorporated into proteins without conversion to Tyr, similar to previous reports that only low levels of turnover of Phe into Tyr were observed when mammalian cells are grown in Tyr-containing culture media^29^. Unexpectedly, the extracellular *T. gondii* tachyzoites which require high energy to empower their gliding motility and invasion into host cells were found incapable of utilizing l-Phe(D8) from culture medium even after 24 hours.

The rapid accumulation of l-Phe(D8) within *T. gondii* supports the role of *T. gondii* in behavioural changes in mice or psychiatric behaviours in infected humans^30^,^31^,^32^,^33^. It has been suggested that *T. gondii* causes these changes *in vivo* and *in vitro* by affecting equilibrium of neurotransmitters, especially dopamine^32^,^34^. This hypothesis has been supported by the discovery of two genes, *AAH1* and *AAH2*, in *T. gondii* genome encoding for two isoforms of aromatic amino acid hydroxylases *AAH* (tyrosine and phenylalanine hydroxylases), which catalyze Phe to Tyr and Tyr to 3,4 dihydroxyphenylalanine (l-Dopa) (the precursor to dopamine), which may alter dopamine pathway, leading to alteration in the behaviour of *T. gondii*-infected host^12^. *AAH1* and *AAH2* differ in their temporal expression*. AAH1* was reported to be constitutively expressed across the parasite life cycle, while the expression of *AAH2* was found to be expressed during the dormant bradyzoite stage^12^. This pattern has led to the assumption that *AAH2* is the candidate effector of the parasite’s manipulation of host dopamine. However, a recent study reported that a ΔAAH2 mutant *T. gondii* strain was not able to increase the production of dopamine in infected mouse brains and that over-expression of the *AAH2* gene could not augment dopamine content *in vitro*^35^. This, to some extent, agrees with our findings that the uptaken l-Phe(D8) was not metabolized into Tyr by the parasite.

This reduction in Tyr during *T. gondii* infection might also have biological effects. Patients suffering from chronic immune activation and inflammatory conditions, which are prominent features in toxoplasmosis, were found to have elevated serum levels of Phe together with elevated Phe to Tyr ratios (Phe/Tyr). These changes in AA levels were associated with neuropsychiatric symptoms, such as depression and mood changes^36^. The rapid uptake of l-Phe(D8) by *T. gondii* indicate the significant need for this AA by *T. gondii* even at an early stage of the intracellular infection cycle. Also, the two predicted *AAH* genes in *T. gondii* reflect the parasite’s ability to internalize and digest Phe from the host^12^. In the present study the amount of l-Phe(D8) uptaken from the host cells and incorporated into protein exceeds the expected amount of new protein formed based on the expected increase in size of the growing *T. gondii* as indicated by the bright field images. It is reasonable to hypothesize that l-Phe(D8) may be transported into *T. gondii* and enter the AA pool of the host cell. This is then exchanged with the original l-Phe residues in its native form and without preliminary degradation similar to the exchange reaction in bacteria^29^.

Another possible mechanism that may facilitate the rapid uptake of l-Phe(D8) is the release of proteins by *T. gondii* into the host cells during the early stage of infection. A range of proteins delivered from rhoptries and dense granules, which are secretory organelles unique to the phylum Apicomplexa, are released by *T. gondii* into the parasitophorous vacuole (PV) within 10 minutes after invasion to subvert host cell function^38^,^39^. Even though the concentration of l-Phe was expected to be very low given the large volume of the mammalian cells, Raman signals corresponding to the 1004 cm^−1^ Raman band, assigned to l-Phe molecules, were detected inside the host cytoplasm at 2–5 hours PI.

*T. gondii* and other protozoans can synthesize few AAs because they have lost key enzymes needed to make some AAs *de novo*. Hence, they rely on host to supply them with AAs, the transport of which is mediated by several plasma membrane carriers (TransportDB). It is very likely that the import of Phe via the plasma membrane and the subsequent entry within the intravacuolar parasites occurs via transporters. Even though AA import is essential for protozoans only a few AA transporters have been functionally characterized, including the AAAP (amino acid/auxin permease) transporter family whose number vary significantly among protozoan species, and may transport one or multiple AAs, reflecting fundamental differences among protozoan species in AA requirements or salvage mechanisms^40^. However, we calculated the transfer rate of l-Phe(D8) molecules from host to *T. gondii* to be 0.5–1.6 × 10^4^ molecules/s. Because an average transporter can have a transport rate of ~10^3^–10^4^ molecules/s, our results agree with the prediction that four AAAP transporters are involved in the AA transfer in *T. gondii* B7 strain (TransportDB). On the other hand, two transport systems for Phe have been identified in the protozoan *Trypanosoma brucei brucei*, characterized by a high and a very high affinity^41^. When the Vmax were expressed in terms of molecules/s/single cell, we estimated transport rates of 9.4 × 10^4^ and 56.4 × 10^4^ of Phe molecules/s for the high and the very high transport system, respectively. These transport rates are 10 to 50-fold higher than the rates for *T. gondii* measured in our study, supporting the idea that differences exist in the trafficking of Phe between different protozoans.

## Conclusions

Common imaging techniques often do not allow distinguishing between newly imported AAs and those existing within the cell, or visualizing individual AA. To overcome these challenges, we used a RMS-based imaging approach to directly monitor the uptake of individual deuterium-labelled Phe by individual *T. gondii* tachyzoites. Using this approach, we showed for the first time that it is possible to follow in real time the uptake and intracellular distribution of l-Phe(D8) within individual live *T. gondii*-infected cells. *T. gondii* tachyzoites acquired l-Phe(D8) from pre-labelled host cells at a very rapid rate (~50% at 2–3 hours PI) and l-Phe became fully substituted with l-Phe(D8) at ~7–9 hours PI. Based on calibration model, it was also possible to estimate the transfer rate of l-Phe(D8) molecules from host to the *T. gondii* to 0.5–1.6 × 10^4^ molecules/s. Although the import of Phe by the host cell plasma membrane has been documented, the transporter(s) responsible of the import of Phe from host cell to the parasite growing within the PV has not yet been identified. Future analyses should address whether disruption of Phe salvage pathway in *T. gondii* may have therapeutic value. Our approach can facilitate the study of other intracellular microbial systems in which metabolic exchanges between the host cell and the intracellular pathogen are prominent.

## Methods

### Reagents

l-Phenylalanine (D8, 98%) (Cat no. DLM-372-1) was purchased from Cambridge Isotope Laboratories, Inc. (Andover, MA, USA) whereas l-Phenylalanine (Cat no. P5482), paraformaldehyde, trypan blue, human serum albumin, and acridine orange were purchased from Sigma Aldrich (St. Louis, MO, USA). Dulbecco’s modified Eagle’s medium low glucose, pyruvate (DMEM, Cat no. 11885) was purchased from Life technologies, Grand Island, NY. Fetal bovine serum (FBS) and penicillin/streptomycin were obtained from Invitrogen Corp., Carlsbad, CA.

### Cell culture

The human retinal pigment epithelial cell line (ARPE-19) was kindly provided by Dr Kevin Webb from University of Nottingham and used at passage 19. As an immune-privileged and easily accessible organ, the eye constitutes a favourable target for *T. gondii*. Hence, we selected this cell line as a model to investigate the molecular changes that accompany *T. gondii* infection to the eye. ARPE-19 cells were maintained in DMEM supplemented with 1% penicillin/streptomycin and 5% heat-inactivated FBS. Cells were incubated in 5% CO2 at 37 °C and were passaged twice weekly. Cell viability was determined by trypan blue exclusion assay and only cell cultures with > 99% viability were used in the experiments.

### Growth and preparation of parasites

Tachyzoites of *Toxoplasma gondii* strain RH were maintained *in vitro* by serial passage on monolayers of human brain microvascular endothelial cells (hBMECs) at 37 °C in 5% CO_2_ as described previously^42^. Infected host cell monolayers were scraped and parasites were isolated from host cells by passage through 25- and 27-gauge needles. Parasites were purified using PD-10 Desalting columns pre-packed with Sephadex G-25 medium as described previously^43^. Purified parasites were centrifuged at 800 × g, washed twice with sterile 1x Phosphate Buffered Saline (PBS; 8 g/l NaCl, 0.2 g/l KCl, 0.2 g/l KH2PO4, 1.15 g/l Na2HPO4), re-suspended in fresh DMEM medium, and quantified using a hemacytometer. The final volume of parasite suspension was adjusted with DMEM to achieve a ratio of 2:1 parasite/host cell for subsequent infection experiments. Parasites’ viability was checked by using trypan blue exclusion assay and parasite suspensions with more than 98% viability were used.

### Stable isotope labelling by amino acids (SILAC)

Amino acid-type selective labelling was achieved by formulating a medium containing all amino acids in unlabelled form except for Phe, which was added with the desired isotope label. To minimize the background signal of the l-Phe, ARPE-19 cells were synchronized overnight in serum-deficient, Phe-free customised DMEM culture medium. The cells were subsequently incubated with SILAC DMEM medium supplemented with various concentrations of Phe(D8). Cells were subjected to Raman imaging analysis daily to determine the time taken by l-Phe(D8) to substitute l-Phe within host cells. The concentration of 66 mg/500 ml of l-Phe(D8) (i.e. twice the concentration of l-Phe present in the normal media) was found to be the lowest concentration that showed a detectable shift of the Phe peak in acquired Raman spectra.

### Infection of host cells with *T. gondii*

ARPE-19 cells (1 × 10^4^ cells/ml) were seeded in titanium cell-chambers, which are fitted in 6-well plastic cell culture plates, with a volume of 2 ml DMEM/chamber. The titanium cell chambers were custom-built with MgF2 coverslips (0.17 mm thick) at the bottom to enable acquisition of Raman spectra. Cells were allowed to grow overnight by incubation at 37 **°C** in a humidified atmosphere of 5% CO_2_ −95% air. Then, cells were serum starved for 18 hours, followed by incubation in l-Phe(D8)-supplemented DMEM for a minimum of 5 doubling times to ensure that cells were uniformly labelled and that l-Phe was completely substituted by l-Phe(D8). Before introducing the parasites to cells, the culture medium was replaced with l-Phe-free media to avoid any false labelling of the parasite with l-Phe(D8) while in the extracellular medium before cell invasion. Parasites are grown in a medium containing l-Phe, but they were transferred to l-Phe-free media before adding them to the cells and during host cell invasion. This avoided introducing l-Phe to l-Phe(D8)-labelled cells and ensured that the only source of Phe to the parasites was from the l-Phe(D8)-labelled host cells. Infection was initiated by adding *T. gondii* tachyzoites at a multiplicity of infection (MOI) of 1 cell to 2 parasites (1C:2P) in 2-ml fresh l-Phe(D8)-free DMEM. The control chambers received only 2-ml DMEM (mock-infected). Culture plates were then incubated to allow infection to progress within ARPE-19 cells. Parasite and control cultures were sampled at 0, 6, 24 and 48 hours post infection (PI). At each sampling time two chambers (one infected and one control) were collected from the 6-well culture plate and subjected to Raman Spectroscopy imaging. At least 10 infected cells were analyzed per chamber. In a separate experiment, we incubated free extracellular tachyzoites that are recovered from l-Phe culture media in l-Phe(D8)-supplemented media. These parasites failed to take l-Phe(D8) from media even after 24 h.

### Time course Raman imaging

Raman spectra were recorded using a home-built confocal Raman micro-spectrometer optimized for live-cell studies. An inverted microscope (IX71, Olympus, Essex, UK) was chosen for the setup as it allows live cell measurement without additional disturbance of objective dipping in culture media required by up-right microscopes. The laser is focused through a quartz coverslip at the bottom of specially designed sample holders. An environmental enclosure (Solent, Segensworth, UK) was integrated to the microscope to maintain suitable culture conditions (37 °C temperature and 5% CO_2_) for living cultured cells. Under these conditions, Raman spectral measurements of individual live cells maintained in their original culture media were obtained over several hours without disturbing the cells while avoiding bacterial contamination. A 785 nm~120 mW (before objective) Ti:sapphire laser (Spectra-Physics, Oxford, UK) was used to excite Raman spectra. The beam diameter was expanded to match the objective pupil. A water-immersion 60x objective, NA 1.20 (Olympus) was used to focus the laser on individual cells as well as for collection of Raman scattered photons. The size of the laser spot was approximately 700 nm on the sample. Light collected by the objective was collimated and focused on a 50 μm diameter optical fibre connected to a spectrometer equipped with a 830 lines/mm grating (spectral resolution of ~1.5 cm^−1^ in the 600–1800 cm^−1^ region) and cooled deep-depletion back-illuminated CCD detector (Andor Technologies, Belfast, UK). Raman spectral imaging was performed by scanning the cells through the laser focus in a raster pattern (1 μm step size) using a high-precision step-motor stage (Prior, Cambridge, UK) and acquiring Raman spectra at each position (1s/pixel). The spectrometer wavenumber axis was calibrated prior to each experiment using tylenol at an accuracy of 0.5 cm^−1^. The raster-scan Raman measurements of live cells at the early stages of invasion (1–12 hours) was limited to areas ranging between 10 μm x 15 μm and 15 μm x 20 μm around the parasites. The imaging time for these measurements was 3–6 min. At the late stages of infection (e.g. 24 and 48 hours), the areas occupied by parasites were larger (up to 30 μm x 30 μm), thus the measurements required 15–20 min.

### Fluorescence staining

Acridine orange (AO) staining was used to distinguish the morphological appearance of the intracellular developing *T. gondii* tachyzoites from host cell organelles. AO is a DNA intercalating dye and is used to differentially stain RNA and DNA inside eukaryotic cells. Cells were fixed in 4% paraformaldehyde for 15 min, followed by washing with 1x PBS twice. Then, cells were incubated with 5% AO staining solution for 10 min. AO fluorescence imaging of *T. gondii*-infected cells was performed on the same cell after completion of the Raman spectral imaging. Imaging was performed by using wide-field fluorescent microscope integrated on the confocal Raman micro-spectrometer. The retrospective positioning and identification of the cells was based on two thin marks engraved on the cell chambers (retro-positioning accuracy was 5 μm).

### Image analysis and data processing

Data pre-processing and analysis was done by using in-house built functions in Matlab (The MathWorks, Natick, MA). Data pre-processing involved cosmic rays removal and background subtraction. Noise reduction was performed using singular value decomposition. The height of each peak of interest was measured after a local linear baseline was subtracted. Human serum albumin (CAS number 70024-90-7, Sigma Aldrich, UK) was used to calibrate the Raman spectrometer (30 Phe residues per albumin molecule).

## Additional Information

**How to cite this article**: Naemat, A. *et al.* Tracing amino acid exchange during host-pathogen interaction by combined stable-isotope time-resolved Raman spectral imaging. *Sci. Rep.* **6**, 20811; doi: 10.1038/srep20811 (2016).
